# Systematic analysis of copy number variants of a large cohort of orofacial cleft patients identifies candidate genes for orofacial clefts

**DOI:** 10.1007/s00439-015-1606-x

**Published:** 2015-11-11

**Authors:** Federica Conte, Martin Oti, Jill Dixon, Carine E. L. Carels, Michele Rubini, Huiqing Zhou

**Affiliations:** Department of Molecular Developmental Biology, Faculty of Science, Radboud Institute for Molecular Life Sciences, Radboud University, Nijmegen, The Netherlands; Medical Genetic Unit, Department of Biomedical and Specialty Surgical Sciences, University of Ferrara, Ferrara, Italy; Faculty of Medical and Human Sciences, University of Manchester, Michael Smith Building, Oxford Road, Manchester, M13 9PT UK; Department of Orthodontics and Craniofacial Biology, Radboud University Medical Center, Nijmegen, The Netherlands; Department of Human Genetics, Radboud Institute for Molecular Life Sciences, Radboud University Medical Center, Nijmegen, The Netherlands

## Abstract

Orofacial clefts (OFCs) represent a large fraction of human birth defects and are one of the most common phenotypes affected by large copy number variants (CNVs). Due to the limited number of CNV patients in individual centers, CNV analyses of a large number of OFC patients are challenging. The present study analyzed 249 genomic deletions and 226 duplications from a cohort of 312 OFC patients reported in two publicly accessible databases of chromosome imbalance and phenotype in humans, DECIPHER and ECARUCA. Genomic regions deleted or duplicated in multiple patients were identified, and genes in these overlapping CNVs were prioritized based on the number of genes encompassed by the region and gene expression in embryonic mouse palate. Our analyses of these overlapping CNVs identified two genes known to be causative for human OFCs, *SATB2* and *MEIS2,* and 12 genes (*DGCR6, FGF2, FRZB, LETM1*, *MAPK3*, *SPRY1*, *THBS1, TSHZ1, TTC28, TULP4, WHSC1, WHSC2*) that are associated with OFC or orofacial development. Additionally, we report 34 deleted and 24 duplicated genes that have not previously been associated with OFCs but are associated with the BMP, MAPK and RAC1 pathways. Statistical analyses show that the high number of overlapping CNVs is not due to random occurrence. The identified genes are not located in highly variable genomic regions in healthy populations and are significantly enriched for genes that are involved in orofacial development. In summary, we report a CNV analysis pipeline of a large cohort of OFC patients and identify novel candidate OFC genes.

**Electronic supplementary material:**

The online version of this article (doi:10.1007/s00439-015-1606-x) contains supplementary material, which is available to authorized users.

## Introduction

Orofacial clefts (OFCs) are characterized by orofacial dysmorphism that may extend from the oral cavity to the whole face, involving also the eyes and ears in the most severe cases. OFCs represent the most common craniofacial malformations and a large fraction of all human birth defects. Collectively, the prevalence of OFCs varies between 1.5 and 25 per 10,000 births worldwide (Mossey and Castilla 2003) depending on geographical location, ethnicity and even socioeconomic status (Murthy and Bhaskar 2009). Although OFCs are repairable surgically with only rare exceptions, such as oblique facial cleft, these defects lead to a wide spectrum of lifelong complications, including feeding difficulties, velopharyngeal insufficiency, speech and acoustic impairment, orthodontic problems, psychosocial issues and economic burden due to medical and behavioral interventions, which greatly affect the quality of life (Mossey et al. 2009; Strauss and Cassell 2009; Wehby and Cassell 2010; Shkoukani et al. 2013). Due to the complex multidisciplinary interventions required to treat the lifelong morbidity associated with OFCs, the costs for these disorders have been estimated to be greater than $100,000 per individual (http://www.cdc.gov). Both the frequency and significant healthcare burdens imposed by OFCs emphasize the need to identify the genetic causes and the molecular and cellular mechanisms of these disorders, which will enable the ultimate aim of improving diagnosis, counseling, care and treatment for affected individuals.

Traditionally, OFCs are classified by phenotypes, based on the severity and the anatomical regions involved. The most common OFC phenotypes are cleft palate (CP), cleft lip (CL) and cleft lip and palate (CLP). However, rare OFCs have also been observed in humans, which may affect other oral structures, such as mandible or lower lip, or even the whole face, including nose, cheeks, eyes and forehead into the hairline. OFCs are generally considered to emerge from disruptions of distinct morphogenic processes which occur at different stages of embryological development (Shkoukani et al. 2013). OFCs are defined as complex multifactorial polygenic traits arising from many etiologies, including single-gene mutations, chromosome aberrations, intrauterine environment anomalies, improper maternal nutrient intake (deficiency of folic acid or zinc, excess of retinoic acid), exposure to teratogens (maternal smoking, alcohol or drug consumption, chemical pollutants), stress, infections, and even parental age and weight during pregnancy seem to contribute to the pathology (Dravet et al. 1992; Abrishamchian et al. 1994; Derijcke et al. 1996; Munger et al. 1996; Abel 1998; Hernández-Diaz et al. 2000; Little et al. 2004; Canfield et al. 2005; Jugessur and Murray 2005; Tamura et al. 2005; Villamor et al. 2008; Mossey et al. 2009; Murthy et al. 2009). The genetic component of OFC etiology is relevant, as demonstrated by the tenfold increased risk that has been observed in monozygotic (40 %) vs dizygotic twins (4.2 %) (Wyszynski et al. 1996).

OFCs can be categorized into syndromic and non-syndromic forms, according to the presence or absence of other cognitive or structural anomalies occurring outside the cleft area in the affected individuals (Cobourne 2004). At least 275 syndromes, whose primary features include OFCs, have been identified but the genetic causes are known only for 75 % of them (Leslie and Marazita 2013). Classical genetic studies of syndromic forms of OFCs have identified causative mutations in genes including *IRF6* [Van der Woude syndrome (OMIM #119300), popliteal pterygium syndrome (OMIM #119500)], *MSX1* [cleft-associated tooth agenesis (OMIM #106600)] and *TP63* [ankyloblepharon-ectodermal dysplasia-clefting (OMIM #106260), ectrodactyly-ectodermal dysplasia-clefting (OMIM #225060)], through linkage analysis, candidate gene approaches and confirmation in studies using animal models (Celli et al. 1999; van den Boogaard et al. 2000; McGrath et al. 2001; Kondo et al. 2002; Dixon et al. 2011; Leslie and Marazita 2013). With the fast development of Next-Generation Sequencing technology, whole exome**-**sequencing studies have identified several causative genes in syndromic forms of OFCs such as *MLL2* [Kabuki syndrome (OMIM #147920)], *DHODH* [Miller syndrome (OMIM #263750)] and *RIPK4* [Bartsocas-Papas syndrome (OMIM #263650)] (Ng et al. 2010a, b; Kalay et al. 2012; Mitchell et al. 2012; Setó-Salvia and Stanier 2014).

The identification of causative sequence variants and the associated genes for non-syndromic OFCs remains challenging, as such cases are often sporadic. Several genes involved in syndromic forms of OFCs have been implicated in non-syndromic OFCs, such as *IRF6* and *MSX1* (Lidral et al. 1998; Van den Boogaard et al. 2000; Jezewski et al. 2003; Suzuki et al. 2004; Vieira et al. 2004; Zucchero et al. 2004; Rahimov et al. 2008; Birnbaum et al. 2009). Genome-wide association studies (GWAS) are often performed to investigate genes and loci contributing to the risk of OFC by statistical analyses. Among genes within ORF loci identified by GWAS, *IRF6* has been demonstrated by various models as the causative gene, whereas the role of other genes, such as *ABCA4* and *MAFB*, remains to be assessed (Beaty et al. 2010).

Large structural alterations of the genome, including deletions and duplications of genomic regions termed copy number variations (CNVs), have been studied in OFC patients using classical genetic analyses such as FISH, CGH arrays or, more recently, SNP arrays (FitzPatrick et al. 2003; Mulatinho et al. 2008; Barber et al. 2013; Izzo et al. 2013). Some of the identified genes including *SUMO*1, *CLPTM1L* and *BMP2* have also been validated in animal models (Shi et al. 2009; Sahoo et al. 2011; Williams et al. 2012). However, due to the relatively low number of OFC patients exhibiting CNVs that are available in individual research centers, a systematic CNV study of a large number of OFC patients has not yet been performed to investigate the etiology of OFCs. Recently, new consortia have been organized to create comprehensive databases of clinical case data by combining the resources from different medical centers worldwide. One of these databases is DECIPHER (Database of Genomic Variation and Phenotype in Humans using Ensembl Resources, https://decipher.sanger.ac.uk/), a database of chromosomal imbalance and phenotype in human with information of more than 25,000 patients, contributed by a consortium of clinical genetic centers and diagnostic laboratories from thirty different countries (Bragin et al. 2014). Similarly, ECARUCA (European Cytogeneticists Association Register of Unbalanced Chromosome Aberrations, http://www.ecaruca.net) is another database that provides both clinical and molecular details on unbalanced chromosomal aberrations, recording over 4800 clinical cases so far (Vulto-van Silfhout et al. 2013). These publicly accessible databases provide rich resources to systematically study genetic mechanisms in a large number of CNV patients with OFCs.

Here, we report a comprehensive bioinformatics and statistical analysis to identify candidate causative genes involved in OFCs by analyzing common CNV regions shared by OFC patients retrieved from DECIPHER and ECARUCA databases. The analysis pipeline includes retrieving patient and CNV data from databases, the identification of overlapping genomic regions and the prioritization of candidate genes in the genomic regions followed by statistical analyses. This study identifies two previously known OFC genes and several novel candidate OFC genes.

## Materials and methods

### Data collection

The patients included in our study were retrieved from two publicly accessible web-based databases of genomic variants and chromosomal aberrations in humans, DECIPHER and ECARUCA, reported till July 2014. Two main criteria were used to select patients: the presence of OFCs, alone or in combination with other phenotypes, and the availability of the CNV location coordinates. Firstly, the terms ‘cleft’ and ‘bifid uvula’ were used to perform the search in the databases, and subsequently, for each identified patient the phenotypes were checked to exclude cases of cleft that do not involve the oral region (e.g., eyelid cleft). To be able to obtain a large number of cases, we decided to include patients with syndromic and non-syndromic OFCs. After selecting the relevant patients, further patient details (ID number, OFCs and other phenotypes, presence of overlapping syndromes) and CNV information (CNV type, size, genomic location in GRCh37/hg19) from ECARUCA and DECIPHER were collected (Supplementary Table 1).

### Identification of overlapping CNV regions

Several Linux BEDtools were used sequentially to identify the deleted or duplicated genomic regions shared among OFC patients (Quinlan and Hall 2010). After sorting based on the genomic locations of CNVs, genomeCoverageBed was run to define the common genomic regions shared by patients’ CNVs, named overlapping regions. For each overlapping region the common genomic sequence (chr:start–end) and the total number of overlaps were analyzed. Subsequently, the BEDtools intersectBed and groupbyBed with the option ‘collapse’ were used in combination to join the BED files of common CNV regions with a list containing patients’ IDs for retrieving the patients who shared deleted or duplicated regions.

For randomization, the shuffleBed command was run repeatedly on the same total number of regions with the same sizes as the corresponding actual deletion and duplication lists to obtain 1000 random permutations. For the random permutations, assembly gaps (telomeres and short arm of chr13, 14, 15, 21, 22), alternative haplotype sequences (e.g., chr6_ssto_hap7) and random contigs (e.g. chr4_gl000193_random) as reported in the UCSC Genome Browser archive (http://hgdownload.soe.ucsc.edu/goldenPath/hg19/database/) were excluded. The same pipeline that was used to identify common deletions and duplications of patients was applied to identify the overlapping regions in each randomized CNV list for the two sets of 1000 randomized lists, termed as randomized overlapping region lists. In each randomized overlapping region list, the mean of the number of the overlaps was calculated, obtaining 1000 means per set, and subsequently the overall mean of the set was calculated based on the 1000 means. To visualize the data, the overall mean and the overall standard deviation were computed with R statistical program (http://www.r-project.org/), and then used to calculate the *z* score of each randomized list given by:

1 $$z_{i} = \left( {\mu_{i} - \overline{\mu } } \right)/\sigma \;\;|\;\;\;i \in R$$

where *μ*_*i*_ represents the mean of overlaps of a specific randomized list (*i*), $\overline{\mu }$ indicates the overall mean (average of all list means) while *σ* states the overall standard deviation. Formula (1) refers to a specific randomized list (*i*), but it was applied to all 1000 elements of the randomization set (*R*). Deletions and duplications were processed separately.

The Shapiro–Wilk test showed that the obtained distribution of the *z* score was not a normal distribution in both deletion and duplication sets, and therefore exact *p* values could not be computed. Instead, empirical *p* values based on counting the number of randomization scores that matched or exceeded the real scores were used. The *z* scores based on the list of overlapping deletions and of overlapping duplications derived from patients’ CNVs were calculated, using the same formula (1) that was applied to the randomized lists.

### Gene retrieval, prioritization and OFC gene enrichment analysis

The UCSC Table Browser (https://genome.ucsc.edu/cgi-bin/hgTables) was used to generate a list of encompassed RefSeq genes for each overlapping region, including not only protein-coding genes but also pseudogenes, miRNAs and long non-coding RNAs (lncRNAs). Gene prioritization was performed based on three criteria: the number of overlapping CNVs (≥2), the number of genes in the overlapping regions (≤5, Supplementary Figure 1) and gene expression levels in mouse embryonic palate detected in an RNA-Seq analysis (nRPK ≥ 59.00, Supplementary Figure 2).

To test whether the prioritized gene list is enriched for known OFC genes, a panel of 126 OFC genes that have been shown to be involved in OFCs or craniofacial development was assembled based on the existing literature, hereafter referred to as OFC-associated genes (OFC-AGs) (Supplementary Table 2). Subsequently, the fold enrichment of the proportion of these OFC-AGs in the prioritized gene list was calculated relative to the proportion of OFC-AGs in all genes retrieved from the deleted or duplicated CNVs. The hypergeometric test was used to evaluate the significance of the enrichment.

### Phenotype mapping

In addition to OFCs, other phenotypes of patients who have deletions or duplications encompassing one or more candidate genes were classified based on the phenotypic feature hierarchy of the Human Phenotype Ontology (HPO) (http://www.human-phenotype-ontology.org/) (Robinson and Mundlos 2010). HPO terms (phenotypic features) annotated to the patients were mapped to the top level of the term hierarchy, which consists of 23 broad phenotypic categories (e.g., abnormality of the nervous system) (Supplementary Table 3). This allows a coarse-grained characterization and comparison of patient phenotypes.

### Generation of RNA-Seq data

All animal experiments were approved by the University of Manchester Ethical Review Committee and performed in accordance with the Animals (Scientific Procedures) Act, 1986, United Kingdom. Matings were established between male and female CD1 mice, the morning of the vaginal plug being counted as E0.5. Microdissected facial processes from E10 and E11 and palatal shelves from E12, E13 and E14 embryos were collected and pooled according to their stages to obtain sufficient amount, and RNA was isolated using the Qiagen RNeasy kit. RNA-Seq libraries were generated using the SOLiDTM Total RNA-Seq Kit. Samples were run on SOLiDTM v4 for single-end 50 bp reads. Poor reads were filtered from the data with SOLiD Preprocess Filter. Reads were mapped to the mouse genome (mm9, NCBI Build 37) using Bowtie 0.12.7 (http://bowtie.cbcb.umd.edu) (Langmead et al. 2009) and assigned to RefSeq transcripts with Partek Genomics Solution (version 6.5, Copyright 2009, Partek Inc., St. Charles, MO, USA). Transcript reads were normalized and differential expression analyzed with DESeq2 (Love et al. 2014). The normalized counts from DESeq2 analysis were then converted to normalized expression value nRPK, by dividing normalized counts by the size (kilobases) of the specific isoform transcript length. A mean of 59.00 nRPK was obtained from expression of all genes and an expression level of ≥59.00 nRPK was set as the cutoff for the candidate gene prioritization (Supplementary Figure 2). The RNA-Seq data are available from ArrayExpress: E-MTAB-3157.

### Analysis of genomic variability score

The DGV (Database of Genomic Variants, http://dgv.tcag.ca/) (MacDonald et al. 2014) dataset was retrieved using the UCSC Table Browser. The human genome (GRCh37/hg19) was divided into windows of fixed size, 1 Mb, using the tool windowBed (Quinlan and Hall 2010), and the numbers of CNVs (observed gains and losses) were summed to obtain the total number of variants in each region. Next, the overall number of structural variants per window was determined using the intersectBed and groupbyBed commands in combination. The windows encompassing assembly gaps (telomeres and short arm of chr13, 14, 15, 21, 22), alternative haplotype sequences (e.g., chr6_ssto_hap7) and random contigs (e.g., chr4_gl000193_random) reported in the UCSC Genome Browser archive (http://hgdownload.soe.ucsc.edu/goldenPath/hg19/database/) were removed to avoid bias. To approximate a normal-like distribution, the logarithmic conversion was applied to the variant counts of the resulting genomic windows. The overall mean (*μ*) of all the count logarithms was computed as well as the overall standard deviation (*σ*) with R statistical program, and subsequently used to calculate the *z* score as follows:

2 $$\mu = \frac{{\sum\nolimits_{i = 1}^{n} {\log_{10} \left( {c_{i} } \right)} }}{n}$$

3 $$\sigma = \sqrt {\frac{{\sum\nolimits_{i = 1}^{n} {(\log_{10} (c_{i} ) - \mu )^{2} } }}{n - 1}}$$

4 $$z_{i} = \frac{{\log_{10} (c_{i} ) - \mu }}{\sigma }\;\;|\;\;\;\;i \in W$$

where log_10_(*c*_*i*_) represents the base-10 log of the count value of structural variants (*c*) in a specific window (*i*) according to DGV, *n* indicates the total number of windows generated from the whole genome (GRCh37/hg19), *μ* is the overall mean (average of the base-10 count logs of all windows) while *σ* is the overall standard deviation. Formula (4) was applied to all the elements of the window set (*W*). In this case, the *z* score was considered as a measure of genome variability.

The genomic windows were intersected with deleted and duplicated regions shared by at least two OFC patients. In case a deleted or duplicated region overlapped multiple windows, the mean of the counts was calculated and then used to determine a single *z* score per region. The variability of known OFC genes and potential candidate OFC genes identified in this work and the variability of OFC-AGs from the literature (Supplementary Table 2) were also evaluated by intersecting their genomic locations with the list of windows. The gene locations were retrieved from the Ensembl database (http://www.ensembl.org/index.html) setting the consistent assembly GRCh37/hg19.

## Results

### Patient cohort

A total of 312 unrelated patients presenting different forms of OFCs (including both syndromic and non-syndromic forms) were analyzed in this study, including 295 retrieved from DECIPHER and 17 from ECARUCA in July 2014 (Table 1). All 312 patients appear to be unique, as the genomic locations of CNVs in these patients are all different (Supplementary Table 1). The most common OFC phenotype in this cohort is CP with 197 patients affected. In addition, there are 41 CLP patients, 30 CL patients, 30 bifid uvula patients and a small number of patients exhibiting minor cleft phenotypes, such as mandible cleft, alveolar ridge cleft and facial cleft (Table 1). In case a minor cleft type was present in combination with main OFC phenotypes, the patient was ascribed to the main OFC group.

**Table 1:** Phenotypes in selected OFC patients from DECIPHER and ECARUCA

Phenotypes	Number of patients	
Cleft lip (CL)	24	CL patients in total 30
CL + Alveolar ridge cleft	3	
CL + Cleft mandible	2	
CL + Cleft lower lip	1	
Cleft lip and palate (CLP)	38	CLP patients in total 41
CLP + Bifid uvula	1	
CLP + Cleft mandible	2	
Cleft palate (CP)	186	CP patients in total 197
CP + Bifid uvula	8	
CP + Facial cleft	1	
CP + Alveolar ridge cleft	1	
CP + Cleft lower lip	1	
Bifid uvula	30	
Oral cleft (unspecified)	10	
Alveolar ridge cleft	2	
Cleft lower lip	1	
Facial cleft	1	
Total	312	

### Identification of overlapping CNV regions in OFC patients

**Fig. 1:**
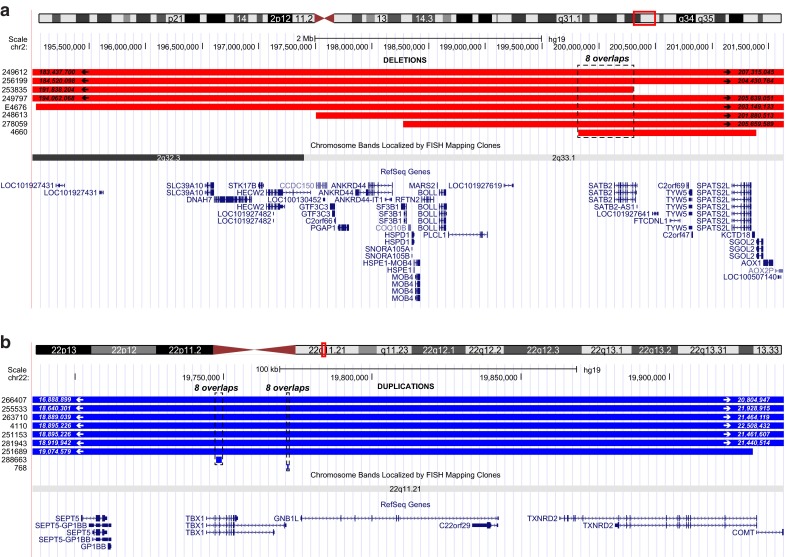
Genomic regions overlapped by eight genomic deletions and by eight genomic duplications in OFC patients. The coordinates of the start and the end of the CNVs are indicated inside the *red*/*blue bars*, if the length of the CNVs are longer than what are shown in the screenshot. The encompassed RefSeq genes and the chromosome ideogram are indicated under the *bars*. a. UCSC Genome Browser screenshot (assembly GRCh37/hg19) of the genomic region on chr2 (0.48 Mb, *dashed box*) overlapped by eight genomic deletions (*red bars*) in OFC patients. b. UCSC Genome Browser screenshot (assembly GRCh37/hg19) of the two genomic regions located on chr22 (1.8 Kb and 1.5 Kb, *dashed boxes*) overlapped by eight genomic duplications (*blue bars*) in eight OFC patients

**Fig. 2:**
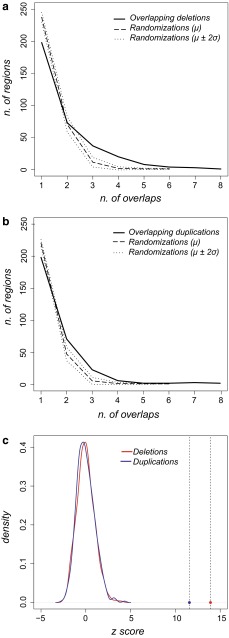
Distribution of overlapping frequencies of CNVs from OFC patients and randomizations. **a** Distributions of the number of overlapping regions of 249 genomic deletions among OFC patients (*solid line*), in comparison to the average number (*μ*, *dashed line*) of the overlapping regions in 1000 randomizations. *σ*, standard deviation. *μ* ± 2*σ*, *dotted lines*. *Y*-axis: number of regions; *X*-axis: number of overlaps. **b** Distribution of the number of overlapping regions of 226 genomic duplications among OFC patients (*solid line*), in comparison to the average number (*μ*, *dashed line*) of overlapping regions in 1000 randomizations. *σ*, standard deviation. *μ* ± 2*σ*, *dotted lines*. *Y* -axis: number of regions; *X*-axis: number of overlaps. **c** The *z* scores of the mean of the overlap frequencies of genomic deletions (*red dot*, *z* = 13.91), and duplications (*blue dot*, *z* = 11.58) are highlighted by *dashed lines*. Kernel density plots on the left side show *z* score distributions of the means of the overlap frequencies from 1000 randomizations based on genomic deletions (*red solid curve*) and on genomic duplications (*blue solid curve*). *Y*-axis: density, *X*-axis: *z* score (based on the overall mean)

To identify the genomic regions that likely contain OFC genes, we identified the genomic CNV regions that are shared by multiple patients. From the cohort of 312 OFC patients with CNVs, 249 genomic deletions and 226 genomic duplications (Supplementary Table 1) were retrieved and analyzed to determine the overlap. Altogether, 146 deletions and 109 duplications that are shared by two or more patients were identified, and these regions are referred as overlapping CNVs hereafter (Table 2). One region of 0.48 Mb located on chr2 is shared among eight OFC patients with deletions, and two regions of 1.8 Kb and 0.5 Kb both on chr22 are shared among eight duplications (Fig. 1). To assess whether the degree of OFC CNV overlap occurs by chance, we performed a randomization analysis (Quinlan and Hall 2010). For the 1000 randomizations based on the deletion list, the maximum overlap number found in the set of randomized regions was 6, and the distribution was uniformly shifted towards lower overlap numbers than that of the OFC deletion list (Fig. 2a). To verify whether this shift is statistically significant, we calculated the *z* scores of the overlap numbers from the deletion list and those from the randomizations. The *z* score of the deletion list is 13.91, markedly higher than those of the randomizations, which are all included in the range −2.55 ≤ *z* ≤ +4.11 (Fig. 2c). As the *z* scores of randomized overlaps do not follow a normal distribution (*p* = 1.38 × 10^−6^, Shapiro–Wilk normality test), we used the conservative empirical *p* value of *p* ≤ 0.001. Similarly, the distribution of randomized overlapping regions based on the duplication list also appeared shifted toward lower numbers, with a maximum overlap number of 6 and a *z* score range of −2.69 ≤ *z* ≤ +4.36 (Fig. 2b). In comparison, the *z* score of the mean overlap number characterizing the OFC duplication list was 11.58, higher than those of the randomizations, with a significant empirical *p* value of *p* ≤ 0.001, as the randomization overlap numbers are not normally distributed (*p* = 1.98 × 10^−8^, Shapiro–Wilk normality test).

**Table 2:** Overview of identified overlapping deletions and duplications

Num. of overlaps	Deletions		Duplications	
	Num. of regions	Length average (bp)	Num. of regions	Length average (bp)
1^a^	198	2242419.58	198	2078953.58
2	73	1808210.63	71	1747913.48
3	37	974799.30	23	471465.30
4	20	1076381.95	6	663154.00
5	8	1186606.63	2	330141.50
6	4	514565.50	2	515747.00
7	3	2487712.67	3	283718.67
8	1	484236.00	2	1177.50

^a^Regions with one overlap represent CNVs that are present in only one patient and are excluded in further analyses

### Candidate gene identification in overlapping CNVs among OFC patients

To identify candidate OFC genes in the CNVs, we searched for genes that are shared by multiple patients and applied a prioritization pipeline. In total, 5809 and 5941 RefSeq genes were retrieved from 249 genomic deletions and 226 genomic duplications, respectively, including protein-coding genes, pseudogenes, miRNAs and long non-coding RNAs. After the gene retrieval, several prioritization criteria were used to identify potential causative genes. Firstly, we excluded genes that are deleted or duplicated in only one patient, resulting in 1651 deleted genes and 1887 duplicated genes. Secondly, the number of genes in the overlapping regions was assessed (Supplementary Figure 1). Among these CNVs, one deleted region covers more than 84 genes and 19 deleted regions contain only one gene. For duplications, one region covers more than 200 genes and in 23 regions only one gene was duplicated. In general, the number of deletions or duplications decreases when the number of their encompassing genes increases. For deletions, there is no clear inflection point at which this number shows a sharp change. Both three and six genes are associated with rather major changes (Supplementary Figure 1a), and deletions with more than six genes fluctuate in frequency at low levels. For duplications on the other hand, there is a clear inflection point at five genes (Supplementary Figure 1b). As the contribution to causality of each gene in regions with a large number of deleted or duplicated genes is difficult to assess, we decided to take a cutoff of the number of genes in the region (≤5 genes, Supplementary Figure 1), and therefore the genes present in regions that contained five genes or fewer were selected. We chose this threshold as it corresponds to a clear inflection point for the duplications and lies between two changing points for the deletions (Supplementary Figure 1). This prioritization step gave rise to 117 deleted and 88 duplicated genes present in the common CNV regions shared by OFC patients (Supplementary Table 4). Subsequently, gene expression in mouse embryonic palatal shelves detected by an RNA-Seq analysis was used as the last prioritization step. To select genes with relatively high expression in developing mouse palates, the distribution of expressed genes (nRPK, normalized reads per kilobase, >0) detected at all analyzed stages (E10-14) was plotted to identify the mean expression level of all genes, which lies at nRPK = 59.00 (Supplementary Figure 2). Finally, the candidate genes with an expression level higher than 59.00 nRPK at any of the five stages (Supplementary Table 4) were selected as potential candidate OFC genes, resulting in 45 genes in deleted CNVs and 27 in duplicated CNVs (Supplementary Table 5).

Among genes in deleted CNVs, two of the genes, *SATB2* and *MEIS2*, which are deleted in eight and five OFC patients (Table 3; Supplementary Tables 2, 5, 6), respectively, have been reported as causative for CP and CLP in several human and animal studies (FitzPatrick et al. 2003; Beaty et al. 2006; Britanova et al. 2006; Dobreva et al. 2006; Erdogan et al. 2007; Leoyklang et al. 2007; Crowley et al. 2010; Johansson et al. 2014; Rainger et al. 2014; Louw et al. 2015). Six additional genes (*THBS1*, *TSHZ1*, *TTC28*, *WHSC1*, *WHSC2* and *LETM1*) encompassed by deleted CNVs have been proposed as the potentially causative genes in critical genomic regions for OFC syndromes (Table 3; Supplementary Tables 2, 5) (Wright et al. 1997, 1999; Stec et al. 1998; Zollino et al. 2000, 2003; Schlickum et al. 2004; Nishiwaki et al. 2006; Coré et al. 2007; Maas et al. 2008; Dostal et al. 2009; Heinonen and Maki 2009; Davidson et al. 2012; Shimizu et al. 2014; Liu et al. 2015). Furthermore, three candidates in deleted CNVs, *FGF2, FRZB* and *SPRY1*, have been shown to contribute to orofacial development in animal models and to be involved with signaling pathways whose disruption leads to OFCs in human (Table 3, Supplementary Tables 2, 5) (Hoang et al. 1996; Lin et al. 1997; Hoang et al. 1998; Mansukhani et al. 2000; Moore et al. 2002; Ignelzi et al. 2003; Sasaki et al. 2006; Szabo-Roger et al. 2008; Dickinson et al. 2009; Porntaveetu et al. 2010; Yang et al. 2010; Kamel et al. 2013). We further identified 34 novel candidates which have not previously been associated with OFCs (Supplementary Table 5). Among 27 genes in duplicated CNVs, three of them, *DGCR6*, *MAPK3* and *TULP4* (Table 3; Supplementary Tables 2, 5), have been previously associated with orofacial development or proposed as causative for OFC syndromes (Demczuk et al. 1996; Lindsay and Baldini 1997; Yamamoto et al. 2003; Singh et al. 2007; Nakamura et al. 2009; Das Chakraborty et al. 2012; Vieira et al. 2015), while the remaining 24 genes are novel candidates (Supplementary Table 5).

**Table 3:** Genes that are identified in overlapping CNVs and have been associated with OFCs and orofacial development

Gene	Overlapping CNVs	Syndromic and non-syndromic OFCs	References
*SATB2*	8 Deletions	Glass Syndrome (OMIM#612313). Isolated CP	FitzPatrick et al. (2003), Beaty et al. (2006), Britanova et al. (2006), Dobreva et al. (2006), Leoyklang et al. (2007), Rainger et al. (2014)
*MEIS2*	5 Deletions	CP associated with cardiac defects and with 15q14 deletion syndrome	Erdogan et al. (2007), Crowley et al. (2010), Johansson et al. (2014), Louw et al. (2015)
*FGF2*	3 Deletions	Involved in craniofacial osteogenesis and suture homeostasis. Ligand of FGFR1 and FGFR2 (known OFC causative genes)	Britto et al. (2002), Sasaki et al. (2006), Szabo-Rogers et al. (2008), Nikopensius et al. (2010), Porntaveetus et al. (2010), Wang et al. (2013)
*FRZB*	2 Deletions	Involved in primary ossification of craniofacial regions via WNT pathway (mouse, zebrafish, xenopus)	Hoang et al. (1996, 1998), Lin et al. (1997). Dickinson and Sive (2009), Kamel et al. (2013)
*LETM1* *WHSC1* *WHSC2 (NELFA)*	3 Deletions	Candidates for Wolf–Hirschhorn syndrome [OMIM #194190]	Wright et al. (1997, 1999), Stec et al. (1998), Zollino et al. (2000, 2003), Schlickum et al. (2004), Maas et al. (2008), Shimizu et al. (2014), Liu et al. (2015)
	3 Deletions		
	3 Deletions		
*SPRY1*	3 Deletions	CP in transgenic mice. Primary paralog of *SPRY2* [Ensembl]	Yang et al. (2010)
*THBS1*	2 Deletions	Craniofacial defects in KO mice. Candidate for 18q deletion syndrome [OMIM#601808]	Nishiwaki et al. (2006), Heinonen and Maki (2009)
*TSHZ1*	6 Deletions	Role in Peters-plus syndrome [OMIM#261540]	Coré et al. (2007), Dostal et al. (2009)
*TTC28*	3 Deletions	Likely gene responsible for Pierre-Robin sequence (including CP) in a case report	Davidson et al. (2012)
*DGCR6*	5 Duplications	Candidate for DiGeorge syndrome [OMIM*601279]	Demczuk et al. (1996), Lindsay and Baldini (1997), Das Chakraborty et al. (2012)
*MAPK3 (ERK1)*	2 Duplications	MAPK pathway involved in craniofacial development. Interactor of *ERK2*, whose disruption leads to OFCs in animal models	Yamamoto et al. (2003), Singh et al. (2007), Nakamura et al. (2009), O’Brien et al. (2015)
*TULP4*	2 Duplications	Statistically significant association in a population-based study	Vieira et al. (2015)

To further test whether the genes identified within CNVs have an enrichment of known OFC genes, we selected a panel of 126 genes, which we termed OFC-associated genes (OFC-AGs), based on the following criteria: (i) genes harboring mutations causing syndromic and non-syndromic OFCs, or (ii) genes located near or within OFC GWAS loci and CNVs, or (iii) genes expressed in lip or palate primordia during development in animal models (Supplementary Table 2). In this panel, 50 genes are shown to be associated with non-syndromic OFCs, 58 are involved in the pathogenesis of syndromes whose features include OFCs, and 18 genes are implicated in both cases. Eleven OFC-AGs are present among the 45 deleted candidates and three among 27 duplicated candidates. This represent a statistically significant 29-fold enrichment (hypergeometric test, *p* = 7.2 × 10^−16^) of OFC-AGs in deleted CNV genes and a 22-fold enrichment (hypergeometric test, *p* = 8.6 × 10^−6^) in duplicated CNV genes (Table 4). Therefore, our data show that the prioritized gene list identified from overlapping CNVs contains a significant number of known OFC-AGs (Table 3).

**Table 4:** Enrichment of OFC-associated genes (OFC-AGs) in candidate OFC genes identified by CNV analysis

	Deletions		Duplications	
	No. of total genes	No. of OFC-AGs^a^	No. of total genes	No. of OFC-AGs
All genes without prioritization	5809	49	5941	30
Prioritized genes	45	11	27	3
Fold enrichment	28.98		22.00	
*P* value (Hypergeometric test)	7.2 × 10^−16^		8.6 × 10^−6^	

^**a**^OFC-AGs: OFC-associated genes. Genes that have been associated with OFC or orofacial development based on extensive literature search (Supplementary Table 2)

**Fig. 3:**
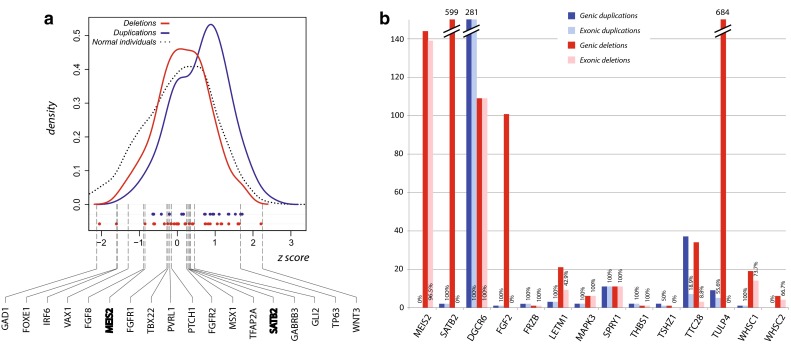
Genomic variability of overlapping deletions and duplications shared by OFC patients and identified candidate genes. **a** Kernel density plot shows variability *z* scores of genomic regions with 1 MB windows in healthy individuals reported in DGV (−5.80 ≤ *z* ≤ + 3.65, *dotted curve*) and of the windows encompassing overlapping deletions (−2.92 ≤ *z* ≤ + 3.02, *red solid curve*) or duplications (−2.30 ≤ *z* ≤ + 3.05, *blue solid curve*) shared by OFC patients. The *dots* on the *X*-axis represent the variability *z* scores of genomic windows encompassing deleted (*red dots*) and duplicated (*blue dots*) candidate genes in CNVs (Supplementary Table 5). The variability *z* scores of windows encompassing 18 OFC-associated genes (OFC-AGs) from the literature are indicated by *dashed lines*. Among these, two genes (*MEIS2*, *SATB2*) highlighted in bold are identified from the overlapping deletions in this study (Table 3; Supplementary Table 2). *Y*-axis: density; *X*-axis: *z* score (variability). **b** The histogram of duplications and deletions at the genomic regions of the 14 known and potential OFC genes identified in healthy individuals, based on DGV dataset (Database of Genomic Variants, http://dgv.tcag.ca/). Duplications in the genic regions of the genes are indicated in *dark blue*, and those affecting exonic regions are indicated in *light blue*. Deletions in genic regions of the genes are indicated in *dark red*, and those affecting exonic regions are indicated in *light red*. *Y-axis* number of CNVs, *X-axis* genes

One interesting question is whether the identified candidate genes are located in highly polymorphic genomic regions, named hypervariable regions (HVRs), in individuals without OFCs. To address this question, we first examined the variability in the whole human genome based on 2,135,523 structural variants from healthy individuals reported in the DGV database (January 2015) (MacDonald et al. 2014) by partitioning the genome to fixed windows and calculating the *z* score of the CNV counts within each window. A total of 2876 1 Mb windows with DGV variant counts were generated and the *z* scores in the healthy population resulted in a range from −5.80 to +3.65 (not normally distributed, Shapiro–Wilk normality test, *p* < 2.2 × 10^−16^) (Fig. 3a). In addition, we assessed the variability of the 126 OFC-AGs from the literature (Supplementary Table 2), and found that they are located within the variability range of the normal population, between −2.84 and +2.30. Next, the *z* scores of the genomic windows encompassing CNVs shared by OFC patients were calculated. The variability distributions of windows encompassing deletions and duplications are in both cases slightly shifted towards increased variability. Nevertheless, they are located within the variability range of the healthy population, from −2.92 to +3.02 for deleted CNVs and from −2.30 to +3.05 for duplicated CNVs but not in HVRs (Fig. 3a). These data show that the OFC CNVs are not located in HVRs in healthy individuals. Next, we focused on the windows containing the identified candidates, 45 deleted genes and 27 duplicated genes, to evaluate the location of those windows in the *z* score distribution. For two deleted (*USP14* and *ZMYND11*) and four duplicated (*RIC8A*, *PSMD13, SIRT3* and *YES1*) novel candidates, the variability score was not assessable due to the exclusion of the windows encompassing telomeric sequences. Importantly, the *z* scores of the other potential OFC candidates lie within the variability range of the normal population, varying between −2.05 (*FAT4*) and +2.21 (*FRG1*) for deleted CNV genes, and between −0.67 (*FIRRE*) to +1.72 (*DGRC6*) (Fig. 3) for duplicated CNV genes, similar to those of the range of the OFC-AGs.

Finally, we assessed the total number of genomic and exonic CNVs in healthy individuals affecting the two known cleft genes in our analysis, *SATB2* and *MEIS2*, and the 12 proposed OFC genes (Fig. 3b). *SATB2* and *MEIS2* appear to be partially deleted in a large number of DGV individuals, 149 and 599, respectively (Fig. 3b; Supplementary Figure 3). Interestingly, all the deletions within *SATB2* in healthy individuals are not located in the exonic regions but map to intronic regions (Fig. 3b; Supplementary Figure 3b), whereas deletions within *MEIS2* disrupt the promoter regions (Fig. 3b; Supplementary Figure 3a). Except for *DGCR6* that is affected by a large number of exonic deletions and duplications, the other eleven genes are not frequently affected by CNVs in healthy population (Fig. 3b). These data suggest that most of the identified candidate genes in OFC CNV patients are not often disrupted in healthy individuals, and therefore the deletion or duplication of these genes likely contributes to the etiology.

Taken together, our data suggest that our systematic large-scale CNV analysis of OFC patients and prioritization of genes in these CNV regions can identify potential candidate OFC genes.

## Discussion

Craniofacial abnormalities including OFCs are among the most significant phenotypes where large CNVs are involved in the etiology (Cooper et al. 2011). However, due to the limited number of OFC patients with CNVs in individual centers, analyses of CNVs from a large number of OFC patients are difficult to perform. In the present study, we analyzed a set of CNVs from a large cohort of OFC patients collected from publicly available CNV databases to identify common genomic deleted or duplicated regions and potential causative genes. Specifically, 312 OFC patients were collected from DECIPHER and ECARUCA databases with a total of 249 genomic deletions and 226 duplications. Overlapping genomic regions shared by patients were analyzed, and RefSeq genes in these regions were prioritized to identify candidate causative genes for OFCs, resulting in 45 potential candidate genes for deletions and 27 for duplications. Furthermore, statistical analyses showed that the overlapping CNV regions in OFC patients are not randomly located, and that the identified candidate genes do not lie within hypervariable genomic regions of the human genome.

Several considerations need to be taken into account with regards to our analysis pipeline for the identification of candidate OFC genes. Firstly, we collected unique OFC patients from DECIPHER and ECARUCA databases, to ensure a single registration of patients and to avoid overrepresentation due to repeated data. Unique registration is required for the submission of CNV patients to the ECARUCA database (personal communication) (de Leeuw et al. 2012). The uniqueness of the collected patients is further confirmed by their different genomic locations (Supplementary Table 1). We note that not all CNVs in these databases may be validated. Secondly, different types of OFC patients including CP, CL and CLP were collected to have a large cohort of patients for statistical analyses, and the analysis pipeline is likely to identify the common mechanisms involved in different OFCs. It should be noted that distinct mechanisms have been proposed for CP, CL and CLP, which likely cannot be distinguished using this approach. Thirdly, we used a cutoff of fewer than five genes in CNV regions to prioritize candidate genes, because it is difficult to evaluate the contribution of each gene when a large number of genes are deleted or duplicated. Given that there are not many CNVs containing more than 5 genes, as compared to smaller CNVs (with ≤5 genes) (Supplementary Figure 1), it is not likely that we miss many OFC relevant genes using this criteria. However, this approach cannot identify genes that have a minor contribution individually but act synergistically when located in the same CNV regions. It is known that one of the intrinsic problems of CNV studies is to identify the dosage-sensitive genes that underlie the phenotypes because CNVs usually encompass many genes (Cooper et al. 2011). Fourthly, one of the strengths in our analysis pipeline is that our study focuses on genes located in overlapping CNVs shared by several patients whose common phenotype is OFC. This strategy allows to identify common genetic mechanisms of OFCs, rather than those of individual patients. Finally, we used gene expression levels in mouse embryonic palatal shelves (Supplementary Figure 2) to prioritize OFC candidate genes. This is based on the hypothesis that genes expressed in embryonic palate are likely involved in the pathogenesis of OFCs. However, some known OFC genes such as *TBX1* that do not have high expression levels in mouse embryonic palates were not selected as the candidate genes using our systematic approach (Supplementary Table 4). *TBX1* is located in the 22q11.2 region that is highly susceptible to rearrangements including microdeletions and microduplications. Patients with 22q11.2 microdeletion syndrome, also known as DiGeorge syndrome [OMIM #188400] or Velocardiofacial syndrome [OMIM #192430], and with 22q11.2 microduplication syndrome often have orofacial abnormalities together with many other phenotypes (Yagi et al. 2003; Torres-Juan et al. 2007; Wentzel et al. 2008). *TBX1* plays a role in early progenitor cells relevant for palate development and is generally considered to be the causative gene in this region (Torres-Juan et al. 2007; Funato et al., 2012; Herman et al. 2012). In our analysis, *TBX1* is present in our duplicated gene list before the prioritization using RNA-seq data (Supplementary Table 4) but is filtered out by this step. This shows that our selection criteria are rather stringent, increasing the probability that identified candidate genes are involved in OFCs. However, OFC genes that are not expressed in embryonic palates might be missed.

Our analysis pipeline identified two known OFC genes, *MEIS2* that is deleted in five CP patients in our analysis and *SATB2* that is deleted in eight CP patients (Table 3; Supplementary Tables 3, 5, 6). Many studies have confirmed the contribution of *SATB2* to CP both in non-syndromic and syndromic forms, causing Glass syndrome (OMIM #612313), Pierre-Robin sequence with or without ankyloglossia and cleft-associated intellectual disability (Supplementary Table 2) (FitzPatrick et al. 2003; Beaty et al. 2006; Britanova et al. 2006; Dobreva et al. 2006; Leoyklang et al. 2007; Rainger et al. 2014). *MEIS2* is one of the recently identified OFC genes, which has been proposed as the main contributor to the pathogenesis of chromosome 15q14 deletion syndrome (Supplementary Table 2) (Erdogan et al. 2007; Crowley et al. 2010; Johansson et al. 2014; Louw et al. 2015). Intriguingly, although these two genes have been classified as OFC causative genes, they appear to be frequently affected by small deletions in healthy individuals (144 deletions in *MEIS2* and 599 deletions in *SATB2*) (Fig. 3b; Supplementary Figure 3). The deletions affecting *SATB2* are all located in intronic regions, suggesting they are not pathogenic in those individuals (Supplementary Figure 3b). In contrast, the majority of the deletions at *MEIS2* (139 out of 144) affect not only introns but also exons and promoters (Supplementary Figure 3a). This raises the question about how ‘healthy’ the normal individuals in the DVG database are. It is known that some of the OFC phenotypes are not directly evident (e.g., submucous CP) and hence difficult to diagnose (Souza et al. 2013). In addition, genetic defects of OFCs are not 100 % penetrant. Therefore, even if small CNVs are found in the gene body regions of potential candidate genes (Table 3; Fig. 3b; Supplementary Figure 3) in ‘healthy’ individual, these genes may still be relevant to OFCs.

As OFCs are often not fully penetrant, we briefly assessed the OFC penetrance in CNVs containing the OFC candidate genes. Indeed, in DECIPHER and ECARUCA databases, many individuals with deleted or duplicated OFC candidate genes show other disease phenotypes but lack an OFC (non-OFC individuals) (Supplementary Table 6). For several deleted genes including *ACBD3*, *FAM98B*, *H3F3A*, *RNPS1*, *SPRED1*, and *THBS1*, the number of non-OFC individuals with deletions of these genes is lower than that of the OFC patients, suggesting that the deletion of these genes might be more penetrant. Furthermore, patients with deletions and duplications of the OFC candidate genes exhibit heterogeneous phenotypes with many other disease features such as defects in the cardiovascular, nervous and skeletal systems (Supplementary Tables 3, 6). A systematic analysis of these additional features may provide insights into novel OFC syndromes.

In addition to the two known OFC causative genes *SATB2* and *MEIS2*, we identified other 12 genes, nine in deletions (*FGF2*, *FRZB,LETM1*, *SPRY1, THBS1*, *TSHZ1*, *TTC28*, *WHSC1*, *WHSC2*) and three in duplications (*DGCR6*, *TULP4* and *MAPK3*), that have been previously proposed as orofacial development regulators or as potential causative genes for OFCs (Table 3; Supplementary Table 5). *WHSC1*, *WHSC2* (aka *NELFA*) and *LETM1* have been proposed to be primarily involved in Wolf–Hirschhorn syndrome (OMIM #194190), whose features include OFC in almost half of the cases (Wright et al. 1997, 1999; Stec et al. 1998; Zollino et al. 2000, 2003; Schlickum et al. 2004; Maas et al. 2008; Shimizu et al. 2014; Liu et al. 2015). Among the OFC patients we collected, all three genes were deleted simultaneously in the same patients, two affected by CP and one by CL (Supplementary Tables 3, 6), consistent with the hypothesis that the deletion of all these three genes is required to cause more severe craniofacial features including OFCs. In a case report describing a patient with a 22q12.2 microdeletion, *TTC28* has been suggested as the likely gene responsible for Pierre-Robin sequence including CP (Davidson et al. 2012). Interestingly, our data show the deletion of *TTC28* in three CP patients (Supplementary Tables 3, 6), which is in agreement with this hypothesis. In human, *TSHZ1* is considered as one of the candidate causative genes for chromosome 18q deletion syndrome (OMIM#601808), which present OFC in 25 % of the cases (Dostal et al. 2009). In addition, knock-out of this gene in mice gives rise to abnormal skeletal morphogenesis and craniofacial defects (Coré et al. 2007). In our cohort, this gene is deleted in two CLP and four CP patients (Supplementary Tables 3, 6). *THBS1* which has been shown to play a role in the etiology of Peters-plus syndrome (OMIM #261540) with OFC as one of the clinical features is deleted in two CP patients (Supplementary Tables 3, 6) (Nishiwaki et al. 2006; Heinonen et al. 2009). Other two genes, *FRZB* and *SPRY1*, found deleted in two and three patients (Supplementary Tables 3, 6), respectively, have been studied in animal models. Specifically, *FRZB* is locally expressed in primary mouth tissues and involved in primary ossification of craniofacial regions by interacting with the WNT pathway (Hoang et al. 1996, 1998; Lin et al. 1997; Dickinson et al. 2009; Kamel et al. 2013). *SPRY1*, characterized by structural and functional similarity with the proposed OFC gene *SPRY2*, has been shown to cause cardiac defects as well as facial cleft and CP in transgenic mice (Supplementary Table 2) (Yang et al. 2010). The Sprouty family proteins inhibit the FGF pathway where several causative OFC genes such as *FGFR1* and *FGFR2* are involved (Supplementary Table 2). Interestingly, one of the main ligands of *FGFR1*/*2*, *FGF2*, was detected as one of the top deleted candidate genes in our analysis (Supplementary Tables 2, 5). Indeed, the role of FGF signaling and *FGF2* in craniofacial development, specifically in osteogenesis and cranial suture homeostasis, has been demonstrated by a number of studies (Mansukhani et al. 2000; Britto et al. 2002; Moore et al. 2002; Ignelzi et al. 2003; Sasaki et al., 2006; Szabo-Rogers et al. 2008; Li et al. 2010; Porntaveetus et al. 2010). In addition, a statistically significant association between FGF2 markers and OFCs has also been reported (Wang et al. 2013; Nikopensius et al. 2010).

Three genes involved in duplications, *DGRC6*, *TULP4* and *MAPK3*, have also been previously proposed to associate with OFC or orofacial development (Supplementary Table 2). *DGCR6*, duplicated in five patients affected by CL, CLP and CP (Supplementary Tables 3, 6), has been proposed in literature to be involved in the developmental defects associated with 22q11.2 deletions syndrome (aka DiGeorge syndrome) (OMIM*601279) (Demczuk et al. 1996; Das Chakraborty et al. 2012). In mouse, it is highly expressed during embryogenesis, probably contributing to neural crest cell migration (Lindsay et al. 1997). A statistically significant association of *TULP4* with OFC has been reported in a recent study based on 6q23.1 fine mapping in a cohort of five hundred OFC patients (Vieira et al. 2015). In our analysis, this gene has been found duplicated in two patients (Supplementary Tables 3, 6). The MAPK/ERK pathway has been shown to be involved in craniofacial development and related diseases (Yamamoto et al. 2003; Singh et al. 2007; Newbern et al. 2008; Nakamura et al. 2009; Parada et al. 2015). Although orofacial defects have been observed in the *Mapk1* (aka *Erk2*) knockout but not in the *Mapk3 (*aka *Erk1*) knockout mice (Newbern et al. 2008; Parada et al. 2015), the cooperation of these two genes has been shown in many cell types and tissues (Srivastava et al. 2011; O’Brien et al. 2015), suggesting a modulating role of *MAPK3* in craniofacial development. In our analysis, *MAPK3* duplication is present in two patients, one affected by CP and the other by CL (Supplementary Tables 3, 6).

In addition to above potential OFC genes, we identified 34 deleted and 24 duplicated genes that have not yet been associated with OFC. These novel candidates cover a broad range of protein types with various functions, such as transcription factors, metabolic enzymes, kinases and phosphatases, structural proteins, signaling mediators, membrane proteins including receptors, and several uncharacterized proteins (Supplementary Table 5). Although each of them has not yet been linked to orofacial development directly, we found that many of these genes are involved in several signaling pathways important for the process, such as RAC1, BMP and MAPK. Some of these genes are involved in multiple pathways, suggesting the combinatory role of these pathways in orofacial development. Specifically, several identified genes are related to the RAC1 pathway. Recent mouse studies suggest the causative role of RAC1 signaling in CP etiology through fibronectin regulation and cytoskeletal reorganization during neural crest cell development and palatal shelf elevation (Thomas et al. 2010; Tang et al. 2015). RAC1 is a ubiquitously expressed Rho small GTPase, inducible by a number of cell-surface receptors and transmembrane adhesion molecules to stimulate different cellular responses, mainly based on actin cytoskeleton remodeling, ROS production and gene expression regulation (Didsbury et al. 1989; Polakis et al. 1989; Ando et al. 1992; Ménard et al. 1992; Ridley et al. 1992; Heyworth et al. 1993; Minden et al. 1995; Westwick et al. 1997; Ridley 2000; Schmitz et al. 2001). Within this pathway, some of our candidate genes are involved in regulating *RAC1* activity, such as *IGF1R*, *FGF2*, *FARP2*, *THBS1*, *YES1* and *SMURF1* (Adams 1995; Adams and Schwartz 2000; Kubo et al. 2002; Pennisi et al. 2002; Jackson et al. 2003; Madura et al. 2003; Wang et al. 2003, 2006; Fera et al. 2004; Shin et al. 2004; Asanuma et al. 2006; Giehl et al. 2008; Kanazawa et al. 2010; Takahashi et al. 2010; Takahashi and Suzuki 2010; Takegahara et al. 2010; Ding et al. 2011; Lee and Kay 2011; He et al. 2013; Deng and Huang 2014; Chatterji et al. 2015). Some of the candidate genes are regulated by *RAC1*, such as *NCKAP1* and *CYFIP1* (Miki et al. 1998; Schenck et al. 2001, 2003; Eden et al. 2002; Billuart and Chelly 2003; Kurisu et al. 2005; Anitei et al. 2010; De Rubeis et al. 2013) and some others are paralogs of *RAC1* interactors such as *PARD3B* and *STK38*, primary paralogs of *PARD3* and *CDC42,* respectively. In addition, other candidates belong to protein families whose members are known to interact with RAC1 pathway, such as *SEPT2* and *SPRY1* (Gross et al. 2001; Yigzaw et al. 2001; Lim et al. 2002; Lee et al. 2004; Poppleton et al. 2004; Nagata and Inagaki 2005; Lito et al. 2009; Ballou et al. 2013; Ireton et al. 2014; Assinder et al. 2015). Related to this pathway, *FAT4* encodes for a cell–cell interaction molecule, a member of the protocadherin family, which has been though to regulate planar cell polarity (Fukata et al. 1999; Kuroda et al. 1999; Evers et al. 2000; Frebourg et al. 2006; Suo et al. 2012; Keeler et al. 2015). *FAT4* is described in OMIM as causative of two non-OFC syndromes, Van Maldergerm syndrome type 2 (OMIM #615546) and Hennekam lymphangiectasia-lymphedema syndrome type 2 (OMIM # 616006). Nevertheless, a 4q deletion syndrome has been characterized in 20 patients, among them four affected by CP and two by CL or CLP (Strehle et al. 2012). The deletions of these six OFC patients encompass nine of our deleted candidate genes including *FAT4* but also *ANKRD50*, *DCTD*, *FGF2*, *FRG1*, *NEIL3*, *SPATA5*, *SPRY1*, *WWC2*, and one duplicated candidate gene, *FAM149A* (Strehle et al. 2012).

Other candidates are involved in BMP signaling include *SMURF1* and *SPRED1*. *SMURF1* is a ubiquitin-protein ligase specific for SMAD proteins in the BMP pathway. It interacts as a negative regulator of BMP signaling pathway and regulates cell motility, signaling and polarity. This interaction with the BMP signaling may be the key point to explain a possible association between *SMURF1* and OFC etiology, as two members of this pathway, *BMP2* and *BMP4*, are already known to be associated with OFCs (Supplementary Table 2) (Zhang et al. 2002; Liu et al. 2005; Marazita 2007; Lin et al. 2008; Suzuki et al. 2009; Suazo et al. 2010; Sahoo et al. 2011; Williams et al. 2012). *SPRED1*, recognized as the causative gene for Legius syndrome (OMIM #611431), interacts with *SPRY2* that has been described as a causative OFC gene (Supplementary Table 2) (Vieira et al. 2005; Goodnough et al. 2007; Welsh et al. 2007; Spurlock et al. 2009; Matsumura et al. 2011; Song et al. 2015). In addition, *SPRED1* and *SPRY2* act as negative regulators of the FGF and MAPK pathways (Katoh and Katoh 2006; Di Bari et al. 2009; Sylvestersen et al. 2011; Zhao et al. 2015), both shown to affect orofacial development in human or in mouse models (Reardorn et al. 1994; Wilkie et al. 1995; Sasaki et al. 2001; Dodé et al. 2003; Yamamoto et al. 2003; Riley and Murray 2007; Singh et al. 2007; Newbern et al. 2008; Nakamura et al. 2009; García-Domínguez et al. 2011).

Same as for *WHSC1*, *WHSC2* and *LETM1*, some of our novel candidates lie in the same duplicated or deleted regions (Supplementary Table 5). The co-localization of these genes in the same CNVs together with their high expression levels in mouse embryonic palate support the hypothesis of a combinatory function. For instance, *NIPA1*, a duplicated novel candidate, whose deletion has been recently confirmed to be pathogenic (Cooper et al. 2011), maps to a duplicated region that contains two other functionally related candidates, *CYFIP1* and *TUBGCP5*.

In conclusion, this study developed a systematic analysis pipeline for the CNV analysis of a large cohort of OFC patients, and for the identification of potential OFC-related genes. As the result, we identified 45 genes in large genomic deletions and 27 in duplications, including several known causative genes for OFC, such as *SATB2* and *MEIS2*. Our study enriches the reservoir of potential causative OFC genes for genetic studies and provides a disease link to many of these genes that are known to be involved in several signaling pathways. Future human mutation analyses and animal model studies are necessary to confirm the role of the identified potential causative OFC genes in OFC-related diseases and in orofacial development.

## Electronic supplementary material

Supplementary material 1 (DOC 47 kb)

Supplementary material 2 (PDF 394 kb)

Supplementary material 3 (XLSX 208 kb)

Supplementary material 4 (XLSX 76 kb)

Supplementary material 5 (XLSX 51 kb)

Supplementary material 6 (XLSX 34 kb)

Supplementary material 7 (XLSX 46 kb)

Supplementary material 8 (XLSX 31 kb)
