# Supplementary material for: Systematic analysis of copy number variants of a large cohort of orofacial cleft patients identifies candidate genes for orofacial clefts
Source: Hum Genet. 2015 Nov 11;135:41–59. doi: 10.1007/s00439-015-1606-x (PMC4698300; doi:10.1007/s00439-015-1606-x)
Supplement: Supplementary file 1 — Supplementary material 1 (DOC 47 kb) [file 439_2015_1606_MOESM1_ESM.doc]

**List of Supplementary Files** submit to *Human Genetics*

**Systematic analysis of copy number variants of a large cohort of orofacial cleft patients identifies candidate genes for orofacial clefts**

Federica Conte^1,2^, Martin Oti^1^, Jill Dixon^3^, Carine EL Carels^4^, Michele Rubini ^2^,*, Huiqing Zhou^1,5^,*.

^1^ Radboud University, Department of Molecular Developmental Biology, Faculty of Science, Radboud Institute for Molecular Life Sciences, Nijmegen, The Netherlands.

^2^ University of Ferrara, Department of Biomedical and Specialty Surgical Sciences, Medical Genetic Unit, Ferrara, Italy.

^3^ Faculty of Medical and Human Sciences, Michael Smith Building, University of Manchester, Oxford Road, Manchester, M13 9PT United Kingdom.

^4^ Radboud university medical center, Department of Orthodontics and Craniofacial Biology, Nijmegen, The Netherlands.

^5^ Radboud university medical center, Department of Human Genetics, Radboud Institute for Molecular Life Sciences, Nijmegen, The Netherlands.

*Co-corresponding author.

**Co-corresponding authors’ contacts**

H. Zhou: tel. *+31 24 3616850* ; e-mail [*jo.zhou@radboudumc.nl*](mailto:jo.zhou@radboudumc.nl)*;* [*j.zhou@science.ru.nl*](mailto:j.zhou@science.ru.nl)

M. Rubini: tel. *+39 0532 974473* ; e-mail [*michele.rubini@unife.it*](mailto:michele.rubini@unife.it)

**Supplementary Tables Captions**

**Supplementary Table 1. Genomic locations of DECIPHER and ECARUCA OFC patients’ CNVs.** Collected from the cohort of 312 OFC patients, the genomic locations of the 249 deletions and those of the 226 duplications are reported in the spreadsheet named *Deletions_249* and *Duplications_226*, respectively. In each table, the first four columns contain details of patients: CNV ID generated using the patient ID (column A, see also spreadsheet *Notes*), cleft phenotype of the patient (column B) and total number of other phenotypes (column C), publications describing the patients (column D). The CNV details are displayed in other six columns as the following: type of CNV (column E), pathogenicity contribution according to the database (column F), cytogenetic band (column G), genomic location and size of the CNV (column H-I).

**Supplementary Table 2. Panel of 126 OFC-associated genes (OFC-AGs) collected from literature search (spreadsheet *OFC-AGs_from_literature_search*) and related references.** For the genes associated with syndromic OFCs (column A), the name of the OFC syndrome and the OMIM ID (columns B-C) are reported as well as the specific cleft type detected in patients (column D). For the genes associated with non-syndromic OFCs in human or in animal models, the type of supportive studies found in the literature are specified (columns F to K) as well as the specific cleft type (column E). The references are indicated with numbers in the spreadsheet named *OFC-AGs_from_literature_search* (columns L to Q), while the complete reference list is reported in the spreadsheet named *References*.

**Supplementary Table 3. Other disease phenotypes of OFC patients with deletions or duplications affecting the candidate OFC genes identified in this study.** The phenotypes of each patient who carries a CNV affecting one or more of the prioritized 45 deleted or 27 duplicated genes were collected and ascribed to the corresponding HPO terms (Human Phenotype Ontology, *www.human-phenotype-ontology.org/*). The extended list of all phenotypes and HPO terms from patients with deletions is reported in spreadsheet *HPO_DEL_patients_extended*, while the list from patients with duplications is reported in spreadsheet *HPO_DUP_patients_extended*. The comparative table containing all the patient phenotypes mapped to the top level of the HPO term hierarchy, which consists of 23 broad phenotypic categories (e.g. abnormality of the nervous system), are reported in spreadsheets *HPO_DEL_patients_Comparison* and *HPO_DUP_patients_Comparison* for patient with deletions and patients with duplications, respectively. The comparative table is organized as the following: patient ID (colum A), database from which the patient was retrieved (column B), cytogenetic band where the CNV is located (column C), presence of other CNVs in the same patient (column D), top candidate genes affected by the CNV (column E), type of OFC of the patient (column F), presence (gray color) of phenotypes mapped to the 23 broad phenotypic categories from HPO (column G to AC). Further details about the OFC phenotypes are described in the spreadsheet named *Notes*.

**Supplementary Table 4. List of candidate genes prioritized based on the analysis pipeline.** The first spreadsheet contains the list of prioritized deleted candidate genes (117) (spreadsheet *Genes_DELETION_analysis_117*). The second spreadsheet contains the list of prioritized duplicated candidate genes (88) (spreadsheet *Genes_DUPLICATION_analysis_88*). The gene name in human (column A), the type of gene (e.g. protein-coding gene) according to GeneCards and Ensembl databases (column B) and the human Ensembl ID (column C) are reported as well as the name of mouse ortholog (column D), the RefSeq transcript ID of the most highly expressed isoform (column E) in embryonic mouse palate (RNA-Seq data) and the levels of expression (nRPK) of the five stage (E10-14) (column F to J).

**Supplementary Table 5.** **List of the top deleted (45) and duplicated (27) candidate genes.** The spreadsheet named *DELETED_candidates_(45)* contains the list of the top candidate OFC genes obtained in deletions after prioritization using RNA-seq data. The spreadsheet named *DUPLICATED_candidates_(27)* contains the list of the top OFC candidate genes obtained in duplications after prioritization using RNA-seq data. Gene symbol, gene name, function description from EntrezGene and UniProt databases, and the OMIM ID of associated syndromes (if present) are reported in the columns A to D. Relevant literature, if present, is listed in column E. The cleft terms used to check the existing publications in PubMed are listed in the spreadsheet named *Notes*. Based on the availability of existing studies, we classified the genes as *known OFC-genes*, *proposed OFC-genes* and *novel candidates* (column F). The number of patients’ CNVs that overlap with the region containing the candidate is indicated in column G. The known OFC-genes sharing the same region of the candidate are shown in column H, while the total number and the symbols of the genes contained in the region are reported in columns I and J. The variability z score in healthy population based on CNVs in healthy population retrieved from DGV database (January 2015) is showed in column M. For two deleted (USP14 and ZMYND11) and four duplicated (RIC8A, PSMD13, SIRT3 and YES1) novel candidates, the variability z score was not evaluated (spreadsheet *Notes*). Gene expression levels (nRPK) in embryonic mouse palate measured at five time-points, E10 to E14, are specified in columns M to Q. The mouse orthologue name and the ID of the most highly expressed isoform (NCBI RefSeq mRNA ID) are defined in column L.

**Supplementary Table 6.** **List of OFC patients and non-OFC individuals carrying CNVs that contain the candidate genes and the phenotypes of the OFC patients (45 deleted genes; 27 duplicated genes).** The list of phenotypes exhibited by the patients carrying deletions that affect candidate genes are listed in the spreadsheet named *Deleted_top_candidates_(45)*. The phenotypes of the patients carrying duplications that affect candidate genes are listed in the spreadsheet named *Duplicated_top_candidates_(27)*. The gene name (column A), the CNV location, the strand (columns B to E) and the number of overlapping CNVs involving the gene (column F) are shown in the table. The number of non-OFC individuals from DECIPHER and ECARUCA, who exhibit CNVs affecting the top candidates but without OFCs, are reported in column G and H, respectively. The total number OFC patients whose CNVs affects the top candidate genes is reported in column I and their IDs in columns J to Q. Beneath each patient ID, the OFC phenotype is reported in bold and flanked by the total number of other (non-OFC) phenotypes exhibited by the patient (in brackets). The color coding, the abbreviations used for indicating the OFC phenotypes and the IDs are explained in the spreadsheet named *Notes*.

**Supplementary Figures Legends**

**Supplementary Figure 1. Distribution of the total number of genes encompassed by deletions and duplications shared among OFC patients.** The linear plots show the distribution of the number of genes encompassed by genomic deletions (A, solid red line) or duplications (B, blue solid line) shared by OFC patients. The chosen cut-off value of included genes (5) is highlighted by dashed line. Y-axis: number of CNVs; X-axis: number of encompassed genes.

**Supplementary Figure 2. Distribution of log_10_(nRPK) representing gene expression in mouse embryonic palates at stages E10-14 detected by RNA-Seq.** The solid line indicates the distribution of gene expression (nRPK > 0) detected in mouse embryonic palates at stages E10-14 using an RNA-Seq analysis, plotted after logarithmic conversion to identify the overall mean, 59.00 nRPK (vertical dashed line). Y-axis: density. X-axis: log_10_(nRPK).

**Supplementary Figure 3. Genomic deletions and duplications affecting SATB2 and MEIS2 in healthy individuals.** Genome Browser screenshots show deletions (observed losses, red bars) and duplications (observed gains, blue bars) in healthy individuals retrieved from DGV database (January 2015*,* http://dgv.tcag.ca/). Each bar representing a CNV region is flanked by the ID of the study where the CNV data are derived (e.g. esv2571731) and the number of the total CNVs reported in that region in the study (in bold, separated by underscore). (A) MEIS2, 144 genomic deletions (139 affecting exons), 0 genomic duplications. (B) SATB2, 599 genomic deletions (all affecting introns only), 2 genomic duplications (both affecting exons).
