# Supplementary figures and images for: Systematic analysis of copy number variants of a large cohort of orofacial cleft patients identifies candidate genes for orofacial clefts
Source: Hum Genet. 2015 Nov 11;135:41–59. doi: 10.1007/s00439-015-1606-x (PMC4698300; doi:10.1007/s00439-015-1606-x)

a

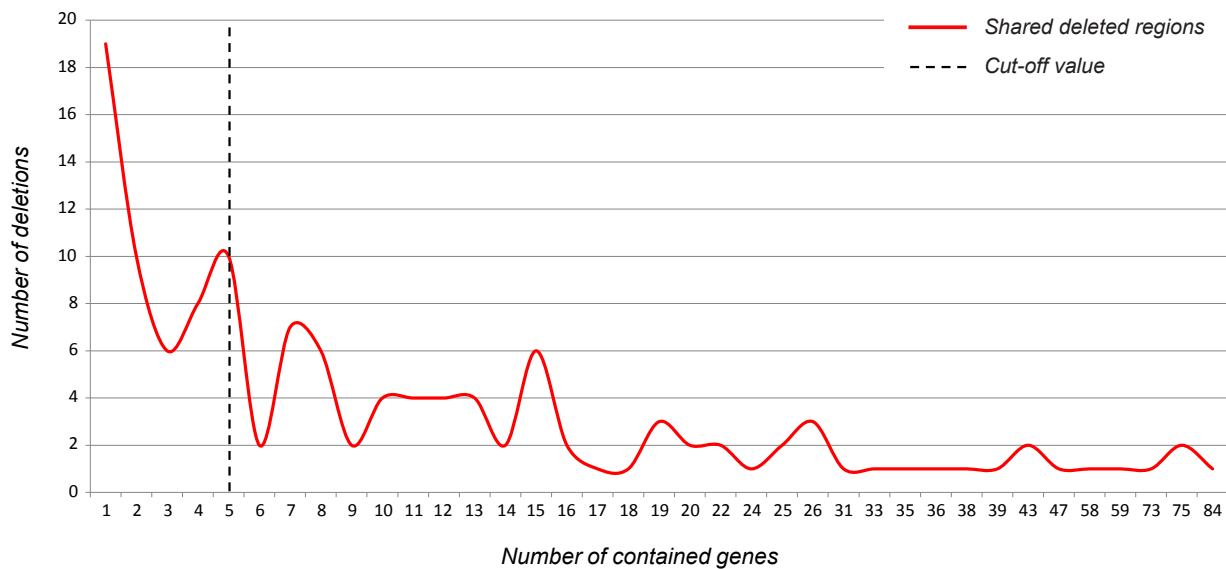

b

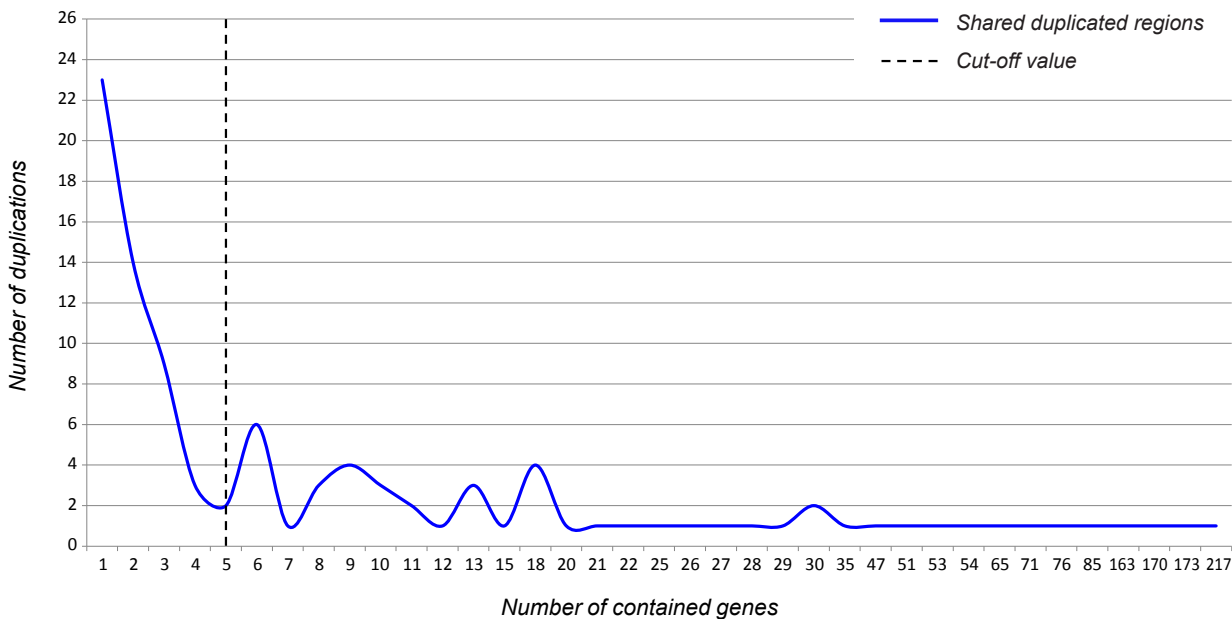

mean = 1.771768

Threshold:  $10^{1.77} = 59.13$  nRPK

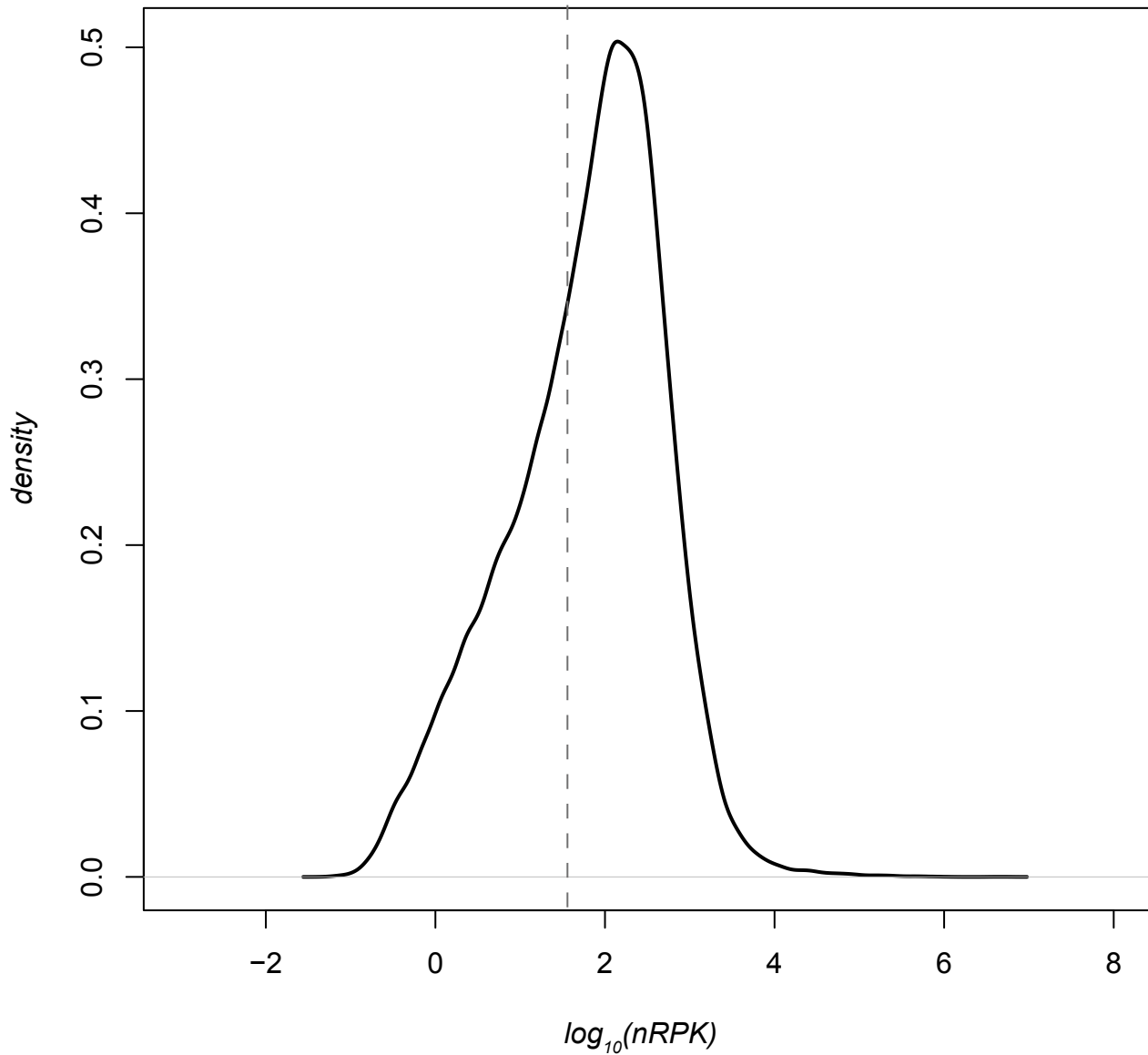

a

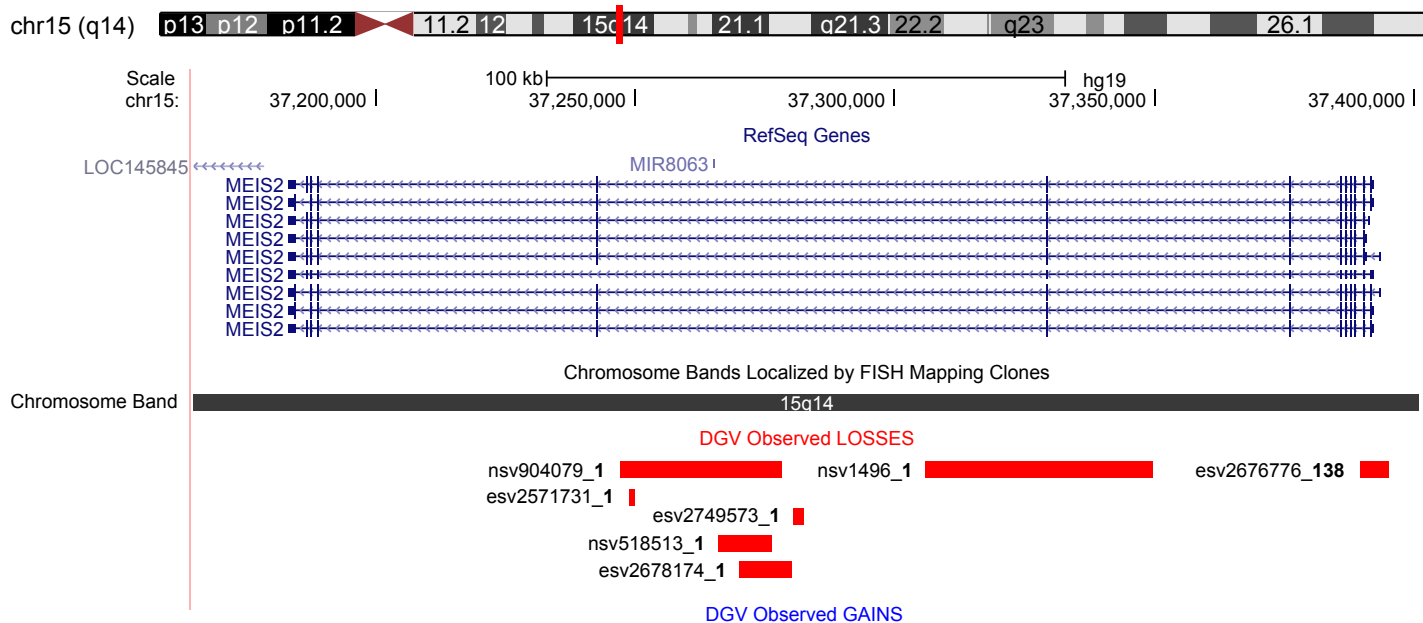

b

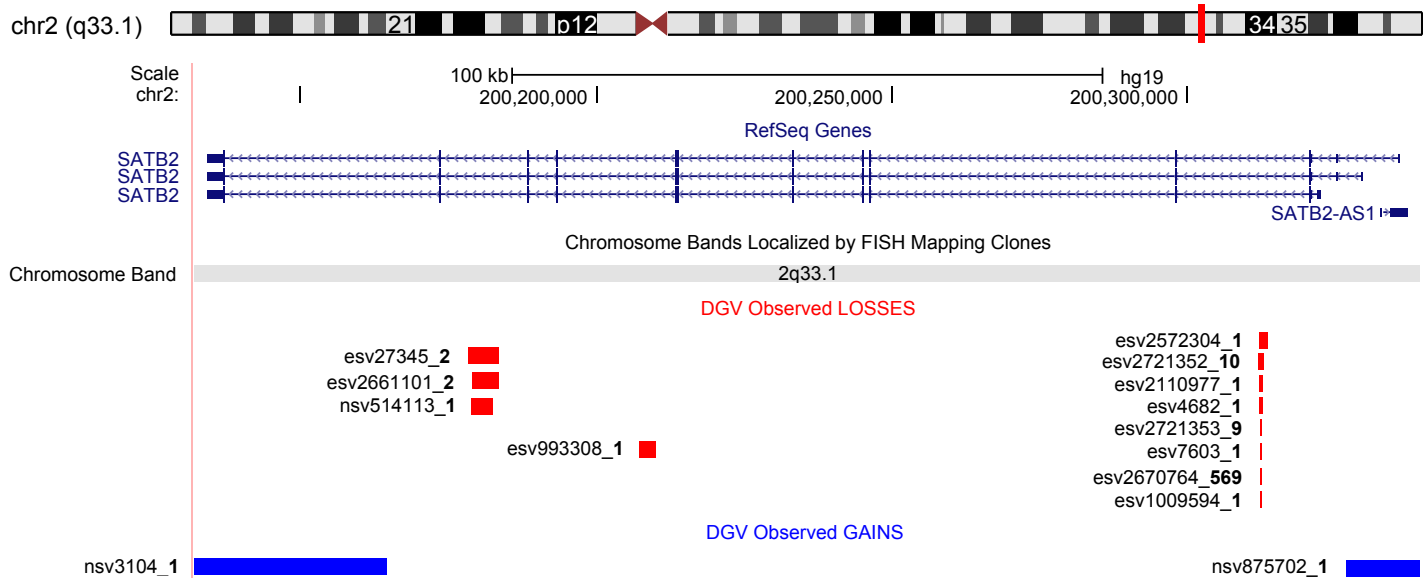

Supplement: Supplementary file 2 — Supplementary material 2 (PDF 394 kb) [file 439_2015_1606_MOESM2_ESM.pdf]
